# IL-6 Receptor Blockade Increases Circulating Adiponectin Levels in People with Obesity: An Explanatory Analysis

**DOI:** 10.3390/metabo11020079

**Published:** 2021-01-29

**Authors:** Stephan Wueest, Eleonora Seelig, Katharina Timper, Mark P. Lyngbaek, Kristian Karstoft, Marc Y. Donath, Helga Ellingsgaard, Daniel Konrad

**Affiliations:** 1 Division of Pediatric Endocrinology and Diabetology, University Children’s Hospital, University of Zurich, CH-8032 Zurich, Switzerland; stephan.wueest@kispi.uzh.ch; 2 Children’s Research Center, University Children’s Hospital, University of Zurich, CH-8032 Zurich, Switzerland; 3 Clinic of Endocrinology, Diabetes and Metabolism, University Hospital Basel, CH-4031 Basel, Switzerland; Eleonora.Seelig@usb.ch (E.S.); katharina.timper@usb.ch (K.T.); marc.donath@usb.ch (M.Y.D.); 4 Center of Inflammation and Metabolism (CIM)/Center for Physical Activity Research (CFAS), Rigshospitalet, University of Copenhagen, DK-2100 Copenhagen, Denmark; mark.lyngbaek@regionh.dk (M.P.L.); Kristian.Karstoft@regionh.dk (K.K.); 5 Department of Clinical Pharmacology, Bispebjerg Hospital, University of Copenhagen, DK-2100 Copenhagen, Denmark; 6 Department Biomedicine, University of Basel, CH-4031 Basel, Switzerland; 7 Zurich Center for Integrative Human Physiology, University of Zurich, CH-8057 Zurich, Switzerland

**Keywords:** adipokine, white adipose tissue, tocilizumab

## Abstract

Human obesity is associated with decreased circulating adiponectin and elevated leptin levels. In vitro experiments and studies in high fat diet (HFD)-fed mice suggest that interleukin-6 (IL-6) may regulate adiponectin and leptin release from white adipose tissue (WAT). Herein, we aimed to investigate whether IL-6 receptor blockade affects the levels of circulating adiponectin and leptin in obese human individuals. To this end, serum samples collected during a multicenter, double-blind clinical trial were analyzed. In the latter study, obese human subjects with or without type 2 diabetes were randomly assigned to recurrent placebo or intravenous tocilizumab (an IL-6 receptor antibody) administration during a 12-week exercise training intervention. Twelve weeks of tocilizumab administration (in combination with exercise training) trend wise enhanced the decrease in circulating leptin levels (−2.7 ± 8.2% in the placebo vs. −20.6 ± 5.6% in tocilizumab, *p* = 0.08) and significantly enhanced the increase in circulating adiponectin (3.4 ± 3.7% in the placebo vs. 27.0 ± 6.6% in tocilizumab, *p* = 0.01). In addition, circulating adiponectin levels were negatively correlated with the homeostatic model assessment of insulin resistance (HOMA-IR), indicating that increased adiponectin levels positively affect insulin sensitivity in people with obesity. In conclusion, IL-6 receptor blockade increases circulating adiponectin levels in people with obesity.

## 1. Introduction

Obesity-associated changes in white adipose tissue (WAT) impact the production and release of adiponectin and leptin, two adipokines mainly produced and secreted by white adipocytes [1]. In fact, human obesity is associated with decreased adiponectin and elevated leptin levels, the latter resulting from an increased release by the expanding fat mass [1]. While adiponectin plays an important role in glucose metabolism by positively affecting insulin sensitivity, leptin is a key hormone in the regulation of body weight, as it reduces food intake and increases energy expenditure [1]. While leptin-induced regulation of food intake and energy expenditure may be impaired in obesity because of emerging leptin resistance, the identification of the pathways that regulate circulating adiponectin and leptin levels is of importance for a better understanding of the derangements of the glucose metabolism and body weight management in people with obesity.

The role of interleukin-6 (IL-6) in the obesity-induced dysregulation of glucose metabolism in adipose tissue is controversial [2]. In human WAT, IL-6 increased leptin production ex vivo [3]. In line with this, we recently revealed that IL-6 signaling in adipocytes contributes to increased circulating leptin levels in obese, but not lean, mice [4]. In fact, high fat diet (HFD)-fed adipocyte-specific glycoprotein 130 knockout (gp130^Δadipo^) mice revealed significantly reduced circulating leptin levels. Of note, gp130 is a mandatory signal transducer protein of the IL-6 signaling pathway, as the IL-6/IL-6 receptor complex interacts with a gp130 homodimer to initiate its signaling. Similarly, exercise trained HFD-fed gp130^Δadipo^ mice revealed lower circulating leptin levels [5], indicating that IL-6 impacts circulating leptin levels in both the sedentary and exercised state. Besides leptin, IL-6 may impact adiponectin production and thus release from white adipocytes [6]. Indeed, IL-6 treatment decreased adiponectin expression in and release from cultured white adipocytes [6]. In support of the inhibitory effect of IL-6 on adiponectin production, HFD-fed IL-6 knockout mice revealed significantly increased circulating adiponectin levels [7]. Taken together, IL-6 may contribute to increased leptin and decreased adiponectin levels in HFD-fed mice. Herein, we aimed to investigate whether the IL-6 receptor blockade affects the serum concentration of these two adipokines in people with obesity.

## 2. Results

**Figure 1:**
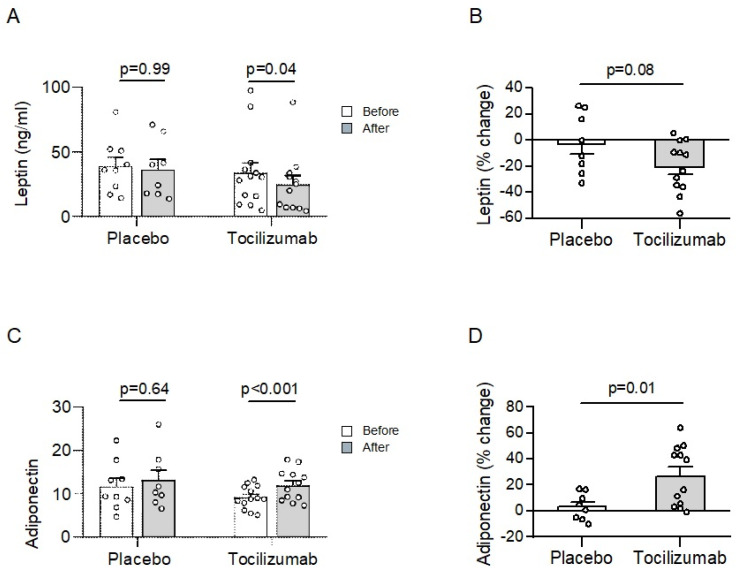
Tocilizumab administration increases adiponectin levels in people with obesity. (**A**,**B**) Serum leptin and (**C**,**D**) adiponectin levels were determined in people with obesity, with or without type 2 diabetes, receiving placebo (*n* = 9) or tocilizumab administration (*n* = 13) before (open bars) and after 12 weeks (grey bars) of administration. Data are displayed as (**A**,**C**) absolute values or as (**B**,**D**) percent change over the 12-week administration period. Values are expressed as mean ± standard error of the mean (SEM).

To investigate whether IL-6 impacts the circulating serum adiponectin and leptin levels in humans, adiponectin and leptin were determined in the serum from people with obesity infused with the IL-6 receptor antibody tocilizumab (*n* = 13) or a placebo (*n* = 9). The baseline characteristics were similar between the treatment groups (Table 1). The results are based on an exploratory analysis of a recently published clinical trial (registration number NCT01073826) [8]. The serum samples were obtained in participants before and after 12 weeks of placebo or tocilizumab administration. Of note, in parallel to the start of the tocilizumab/placebo administration, study participants performed a similar number of exercise training sessions per week (Figure S1A) throughout the 12-week intervention period, leading to non-significant changes in body weight (Figure S1B). However, the infusion of tocilizumab in combination with exercise training for 12 weeks significantly decreased the circulating leptin levels in people with obesity (Figure 1A). In contrast, the leptin levels did not significantly change in the placebo group. Moreover, the decline in circulating leptin was ~8-fold higher in individuals treated with tocilizumab (Figure 1B). Such a difference revealed a statistical trend towards significance.

Next, we assessed the effect of IL-6 on circulating adiponectin levels. As depicted in Figure 1C, adiponectin levels significantly increased after 12 weeks in the tocilizumab-treated group, but not in the placebo-treated group. Accordingly, the increase in circulating adiponectin levels was significantly enhanced in people with obesity treated with tocilizumab (Figure 1D).

**Figure 2:**
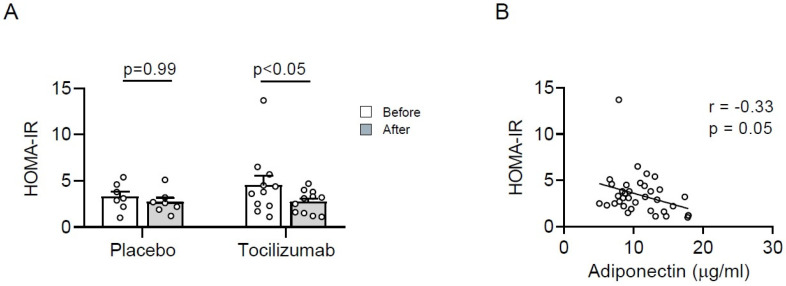
Tocilizumab administration decreases HOMA-IR in people with obesity. HOMA-IR was determined in people with obesity, with or without type 2 diabetes, receiving placebo (*n* = 7) or tocilizumab administration (*n* = 11) before (open bars) and after 12 weeks (grey bars) of administration. Data are displayed as absolute values (**A**) over the 12-week administration period. Values are expressed as mean ± SEM. (**B**) Scatter plot and correlation coefficient (r) of circulating adiponectin concentration and HOMA-IR before and after 12 weeks of administration in people with obesity, with or without type 2 diabetes, receiving placebo or tocilizumab.

In order to investigate whether increased adiponectin levels after tocilizumab administration positively affect insulin sensitivity, the homeostatic model assessment of insulin resistance (HOMA-IR) was assessed. Infusion of tocilizumab but not placebo, in combination with exercise training for 12 weeks, significantly decreased HOMA-IR (Figure 2A). Moreover, circulating adiponectin levels were negatively correlated with HOMA-IR (Figure 2B), indicating that increased adiponectin levels positively affect insulin sensitivity, as expected.

## 3. Discussion

This exploratory analysis revealed that long-term IL-6 receptor blockade (using tocilizumab) in combination with exercise training impacts on the serum adiponectin levels in people with obesity. The administration of tocilizumab during a 12-week training period led to an ~8-fold enhanced increase in adiponectin levels (*p* = 0.01) in people with obesity, with or without type 2 diabetes, compared with the placebo-treated subjects. Furthermore, tocilizumab trend wise enhanced the decrease in circulating leptin levels. As adiponectin is mainly produced and secreted by white adipocytes [1], these data suggest that IL-6 signaling in adipocytes contributes to reduced circulating adiponectin leptin levels in people with obesity. As adiponectin has insulin-sensitizing properties, IL-6 receptor blockade in combination with exercise training may improve adipocyte function, thereby reducing the risk of developing obesity-associated glucose intolerance. Indeed, tocilizumab, but not placebo administration, reduced HOMA-IR herein, and HOMA-IR was negatively correlated with circulating adiponectin levels.

That the IL-6 receptor blockade may have a positive effect on adipocytes in people with obesity is somewhat contradictory to its effects on adipose tissue mass [9,10]. We recently observed that exercise-induced reductions in visceral and epicardial adipose tissues were completely abolished in the presence of IL-6 receptor blockade in people with obesity [9], suggesting that IL-6 may play a central role in regulating adipose tissue mass expansion in humans. Despite these effects on adipose tissue mass, there was no significant change in adiponectin levels in this study [10]. Of note, participants performed supervised high-intensity interval training in the latter study [9,10], whereas in the current study, they performed unsupervised continuous training [8], potentially explaining the divergent findings. However, in line with the data presented herein, tocilizumab significantly increased the serum adiponectin levels in patients with rheumatoid disease [11,12]. Of note, no placebo controls were included in the latter studies.

The limitations of this study are the low number of analyzed samples, as well as the fact that 6 of the 22 included people with obesity suffered from type 2 diabetes. While diabetes status may impact on circulating adipokine levels, the low number of analyzed samples does not allow for performing a sub-group analysis. Moreover, analyzed samples were obtained during a dual intervention (exercise and IL-6 receptor blockade), both of which may affect circulating adipokine levels. In fact, exercise training in people with obesity is associated with increased circulating adiponectin and reduced leptin levels [13]. In the present study, we did not observe significant changes in the circulating adiponectin and leptin levels after 12 weeks of exercise training in the placebo group.

Taken together, tocilizumab administration in combination with exercise training increased the circulating adiponectin levels in people with obesity. In turn, elevated adiponectin levels after tocilizumab administration may improve glucose metabolism.

## 4. Materials and Methods

### 4.1. Study Design and Participants

The reported results are exploratory and from a randomized, placebo-controlled, double-blind multicenter study [8]. All of the participants had a screening visit (V1), followed by five study visits (V2–V6). Placebo or intravenous tocilizumab (8 mg/kg body weight; RoActemra, Roche Pharma AG) administration was randomly assigned and administered at V3 (week 0), V4 (week 4), and V5 (week 8). Tocilizumab was used to block IL-6 receptor signaling, as the aim of the clinical study was to investigate the role of IL-6 in beta cell adaptations during exercise training. The chosen tocilizumab dose is the same as that used when treating patients with rheumatoid arthritis [14]. Baseline sampling was performed at V2. The last blood sampling was performed at V6 (week 12). Twenty-four people with obesity (BMI > 30), with (*n* = 7) or without (*n* = 17) type 2 diabetes, were allocated to tocilizumab (*n* = 15) or placebo (*n* = 9) administration. Unsupervised exercise training started after V3, consisting of bicycle training with a 5 min warm up, followed by 45 min at 75% VO_2max_ ~3 times a week. Adherence to exercise training was monitored through the use of heart rate monitors and training diaries. Study participants came to the hospital every 4 weeks for drug supply (infusion of saline or tocilizumab) and to monitor compliance. 

### 4.2. Measurement of Serum Adiponectin and Leptin

The serum was prepared from blood collected after an overnight fast at V2 and V6. Circulating adipokines were determined in 13 tocilizumab- and 9 placebo-treated subjects (Figure S2). Two participants in the tocilizumab group were excluded because of a lack of training compliance. For some participants, the serum was not available for both analyzed time points. Leptin was determined using a human leptin Mesoscale Discovery (MSD; Rockville, MD, USA) assay K151BYC-2, and the serum adiponectin was measured using MSD assay K151BXC-2.

### 4.3. HOMA-IR

The HOMA-IR was determined as previously described [8].

### 4.4. Statistics

A power analysis was performed related to changes in the glucagon-like peptide 1 levels, but not related to adiponectin or leptin [8]. Data are expressed as mean ± standard error of the mean (SEM). Data were analyzed by linear regression, mixed-effects analysis with Bonferroni’s multiple comparisons test when comparing two groups at two time points, or two-tailed Student’s *t*-test. Statistical tests were performed using GraphPad Prism 8 (GraphPad Software, San Diego, CA, USA). *p* values < 0.05 were considered statistically significant.

## Figures and Tables

**Table 1:** Baseline characteristics.

Characteristic	Placebo	Tocilizumab
Age (years)	45.4 (±4.9)	44.5 (±4.6)
Female sex (%)	77.8	38.5
BMI (kg/m^2^)	32.6 (±0.7)	35.7 (±1.1)
IL-6 (pg/mL)	0.6 (±0.1)	0.8 (±0.2)
hs-CRP (nmol/L)	3.3 (±0.8)	2.5 (±0.5)
Total cholesterol (mmol/L)	4.5 (±0.3)	4.5 (±0.2)
Triacylglycerol (mmol/L)	1.3 (±0.2)	2.0 (±0.3)

## Data Availability

The data supporting the findings of this study are available within the article or from the corresponding authors upon reasonable request.

## Supplementary Materials

The following are available online at https://www.mdpi.com/2218-1989/11/2/79/s1. Figure S1, (**A**) Number of performed exercise training sessions per week in people with obesity with or without type 2 diabetes receiving placebo (*n* = 9) or tocilizumab administration (*n* = 13). (**B**) Body weights in people with obesity with or without diabetes receiving placebo (*n* = 9) or tocilizumab administration (*n* = 13) before (open bars) and after 12 weeks (grey bars) of administration.
