# Supplementary material for: IL-6 Receptor Blockade Increases Circulating Adiponectin Levels in People with Obesity: An Explanatory Analysis
Source: Metabolites. 2021 Jan 29;11(2):79. doi: 10.3390/metabo11020079 (PMC7911215; doi:10.3390/metabo11020079)
Supplement: Supplementary file 1 [file metabolites-11-00079-s001.zip › metabolites-1066656-supplementary.pptx]

## Slide 1
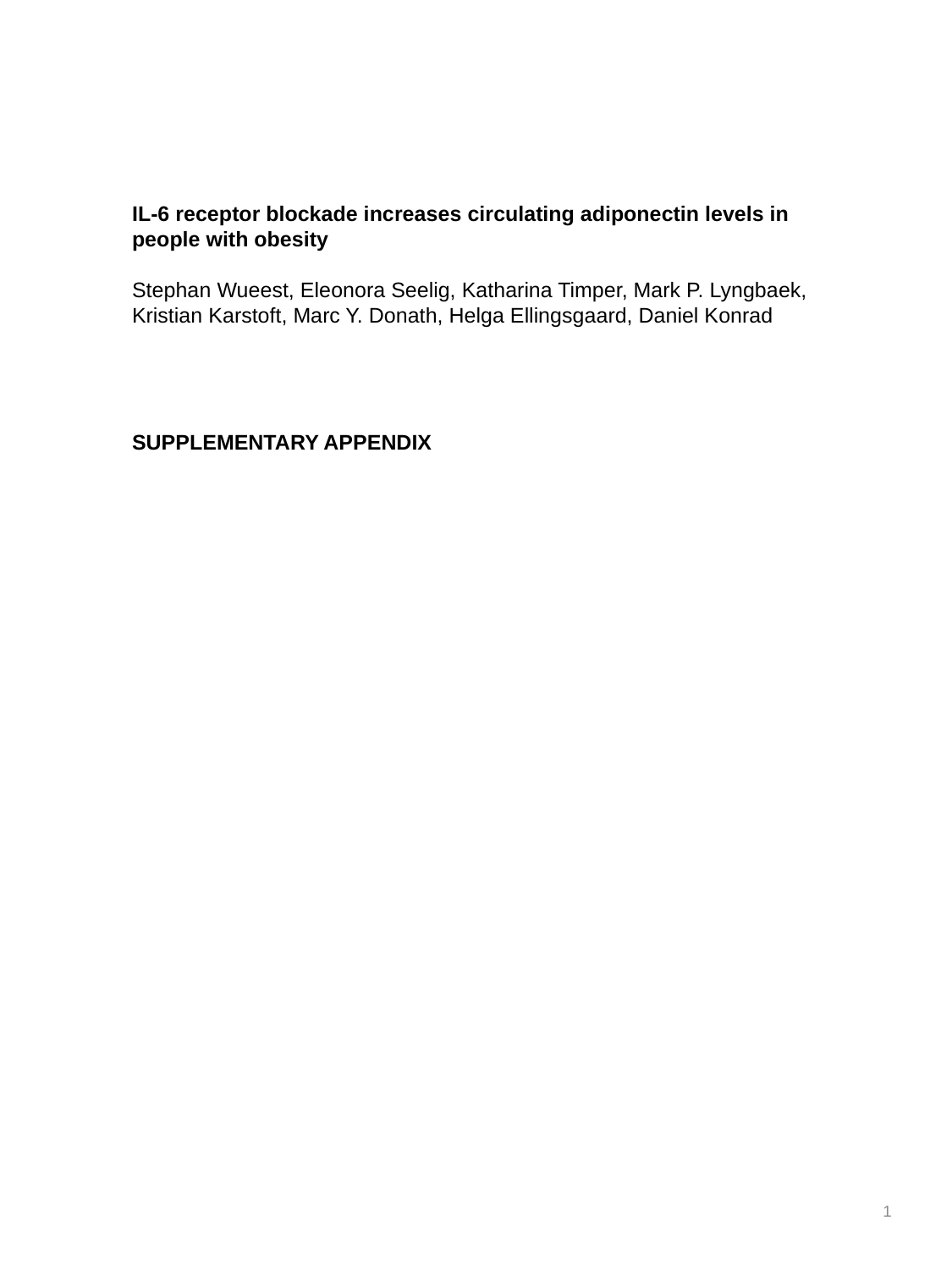

IL-6 receptor blockade increases circulating adiponectin levels in people with obesity
Stephan Wueest, Eleonora Seelig, Katharina Timper, Mark P. Lyngbaek, Kristian Karstoft, Marc Y. Donath, Helga Ellingsgaard, Daniel Konrad
SUPPLEMENTARY APPENDIX
1

## Slide 2
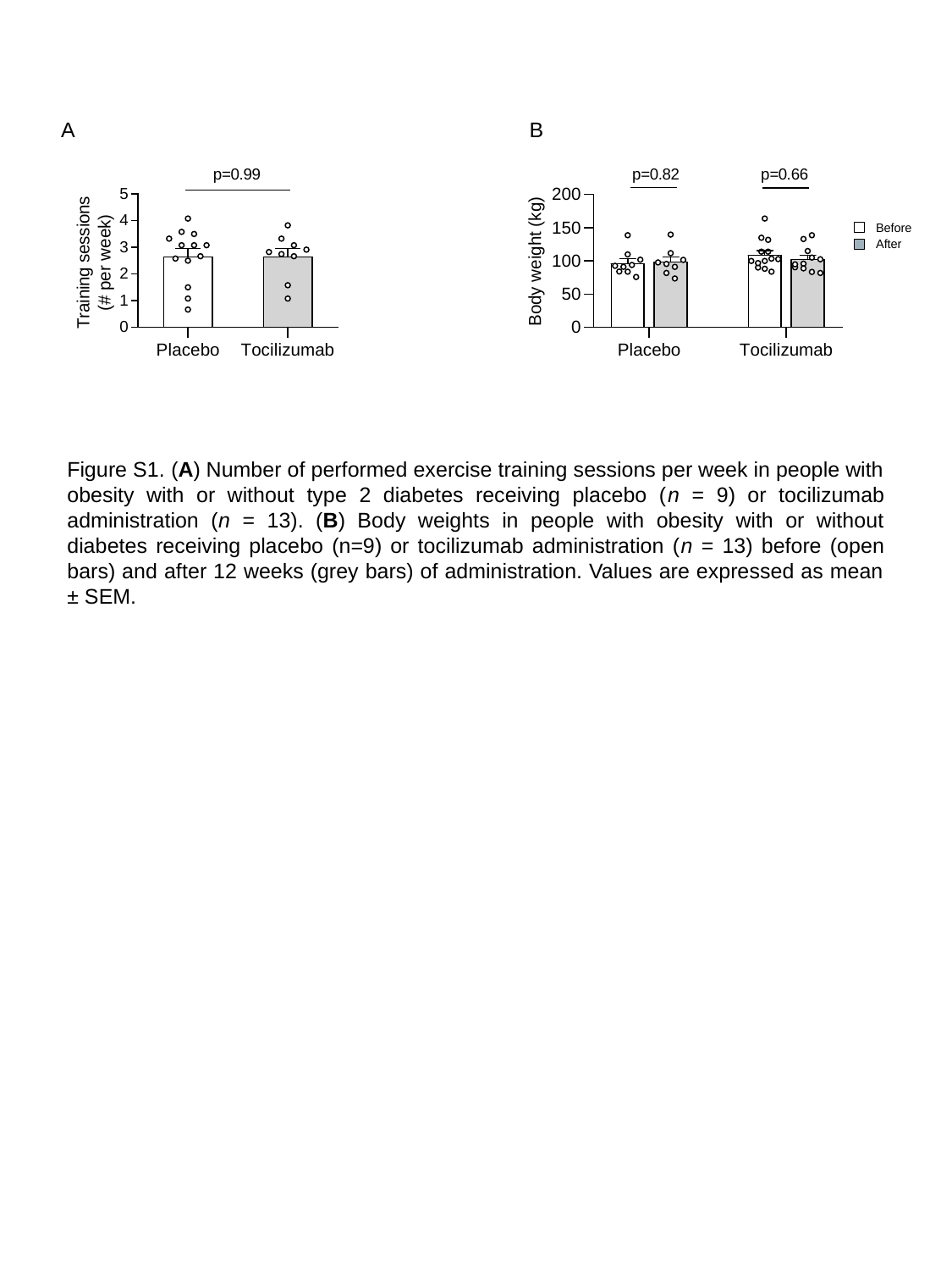

A
B
Before
After
Figure S1. (A) Number of performed exercise training sessions per week in people with obesity with or without type 2 diabetes receiving placebo (n = 9) or tocilizumab administration (n = 13). (B) Body weights in people with obesity with or without diabetes receiving placebo (n=9) or tocilizumab administration (n = 13) before (open bars) and after 12 weeks (grey bars) of administration. Values are expressed as mean ± SEM.

## Slide 3
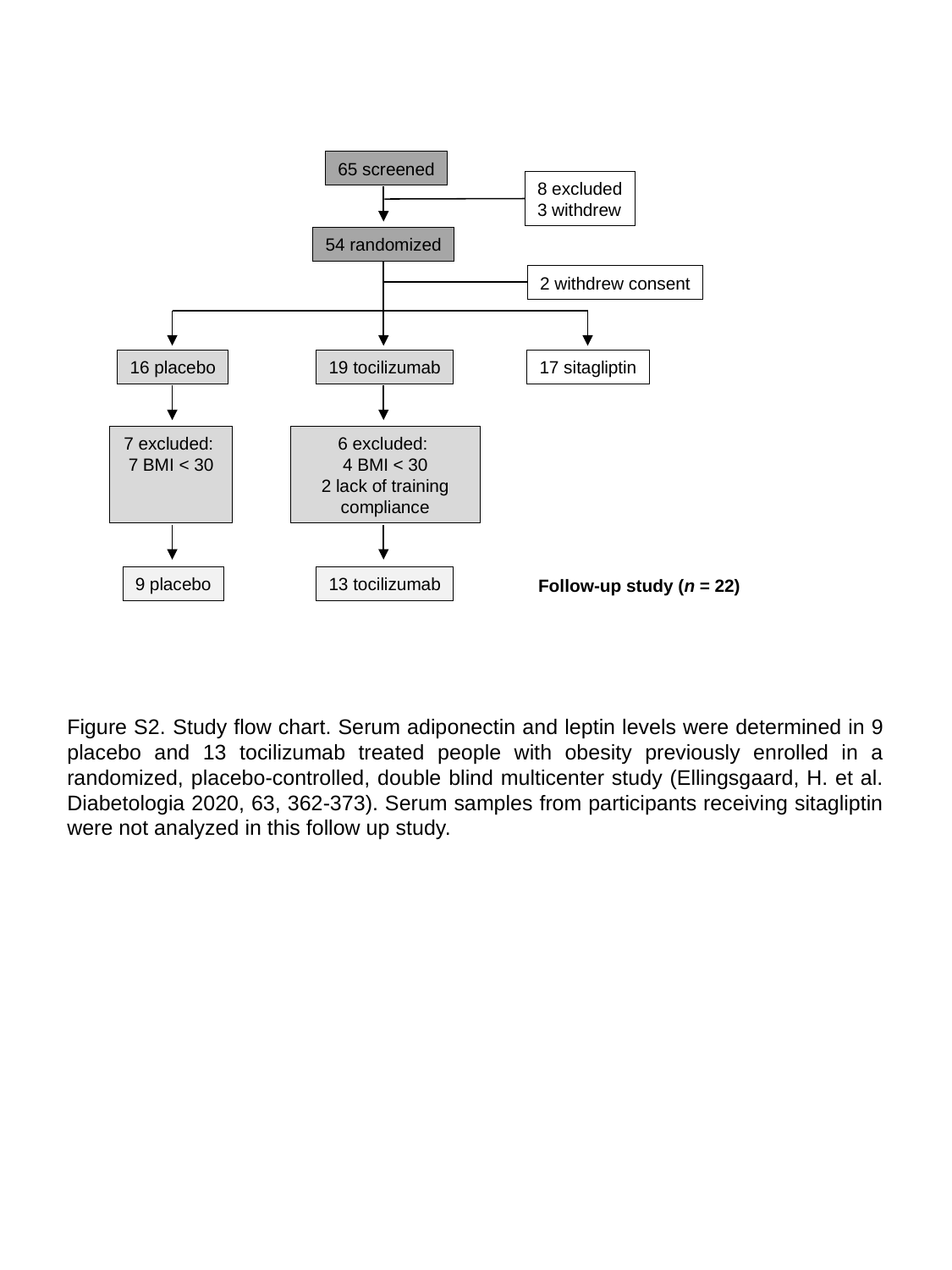

65 screened
8 excluded
3 withdrew
54 randomized
2 withdrew consent
16 placebo
19 tocilizumab
17 sitagliptin
7 excluded:
7 BMI < 30
6 excluded:
4 BMI < 30
2 lack of training compliance
9 placebo
13 tocilizumab
Follow-up study (n = 22)
Figure S2. Study flow chart. Serum adiponectin and leptin levels were determined in 9 placebo and 13 tocilizumab treated people with obesity previously enrolled in a randomized, placebo-controlled, double blind multicenter study (Ellingsgaard, H. et al. Diabetologia 2020, 63, 362-373). Serum samples from participants receiving sitagliptin were not analyzed in this follow up study.
